# *Artemisia rupestris* Improves the Microbiota–Barrier Axis in DSS Colitis

**DOI:** 10.3390/ijms27146478

**Published:** 2026-07-21

**Authors:** Jiaying Wu, Xuwen Mao

**Affiliations:** 1 College of Pharmacy, Xinjiang Medical University, Urumqi 830017, China; 17699941998@163.com; 2 Xinjiang Key Laboratory of Biopharmaceuticals and Medical Devices, Xinjiang Medical University, Urumqi 830017, China; 3 Xinjiang Key Laboratory of Natural Medicines Active Components and Drug Release Technology, Xinjiang Medical University, Urumqi 830017, China

**Keywords:** *Artemisia rupestris* water extract, inflammatory bowel disease, sodium dextran sulfate-induced colitis, gut microbiota, non-targeted metabolomics

## Abstract

Gut microbiota dysbiosis and metabolic disorders are key factors in inflammatory Bowel Disease (IBD) pathogenesis and progression. Although *Artemisia rupestris* water extract (AR) contains many anti-inflammatory compounds, its effects and mechanisms in colitis have not yet been explored. This study is the first to evaluate AR’s therapeutic efficacy in dextran sodium sulfate (DSS)-induced colitis and to investigate whether AR acts by modifying the gut microbiota and correcting metabolic imbalances. An acute inflammatory bowel disease model was established in mice using 3% DSS. Mice received AR at different doses, and clinical and pathological indices, histopathological scores, and blood concentrations of pro-inflammatory cytokines were evaluated. We explored AR; mechanisms in IBD using 16S rRNA sequencing and untargeted metabolomics. AR decreased concentrations of pro-inflammatory cytokines IL-6, CXCL-1, and TNF-α, and reduced MPO levels. It reduced intestinal permeability and mitigated IBD symptoms. Immunohistochemistry demonstrated that AR downregulated TLR4 and TLR9, while restoring Occludin (OCC) and Zonula Occludens-1 (ZO-1). AR modified the gut microbiota by reducing aberrant taxa such as *HT002* and *Erysipelatoclostridium*, while increasing beneficial bacteria including *Bacteroides*, *Alloprevotella*, and *Lachnospiraceae_NK4A136_group*. AR also rectified metabolic disturbances by reducing 5OH-HIP, reversing decreases in metabolites such as 4-(2-amino-3-hydroxyphenyl)-2,4-dioxobutanoate and vanillin, and influencing amino acid and lipid metabolic pathways. AR treatment produces therapeutic effects distinct from other interventions by targeting inflammatory responses, restoring the gut microbiota and metabolism, suppressing TLR4 and TLR9 signaling, and repairing the mucosal barrier.

## 1. Introduction

Inflammatory bowel disease (IBD) is a chronic condition marked by ongoing or recurrent inflammation of the gastrointestinal tract. Increasing data indicate that the progression of IBD is influenced by multiple factors, including genetic predisposition, immunological dysregulation, environmental influences, and dysbiosis of gut microbiota [1]. In recent years, the global prevalence of IBD has continued to rise, with increasing incidence reported not only in traditional high-prevalence regions but also in developing countries [2,3]. Although conventional medications can control and alleviate symptoms to some extent, long-term use may lead to drug dependence and adverse reactions [4]. Accordingly, identifying more effective therapeutic agents remains of considerable importance.

Accumulating evidence indicates that traditional Chinese medicine can modulate gut microbial composition and intestinal barrier integrity through multi-component, multi-target actions, thereby alleviating IBD inflammation and improving disease phenotypes [5,6,7]. *Artemisia rupestris* (AR; *Artemisia rupestris* L.) is the whole herb of a plant belonging to the genus Artemisia (Asteraceae). Modern pharmacological studies indicate that AR extracts exhibit multiple bioactivities, including anti-inflammatory, hepatoprotective, antioxidant, and antitumor effects [8,9], which are closely related to major pathological processes of IBD. AR extract is rich in diverse chemical constituents, primarily flavonoids, sesquiterpenes, volatile oils, and polysaccharides [10]. Among these, flavonoids (e.g., luteolin and quercetin) and sesquiterpenes (e.g., artemisinic acid) are considered closely associated with its pharmacological activity [11,12]. Research indicates that luteolin reduces the release of proinflammatory mediators by obstructing the activation of the NF-κB and ERK channels of signaling [13], whereas quercetin displays anti-inflammatory effects by suppressing the TLR4/NF-κB pathway [14]. Additionally, aqueous extracts of AR have been reported to possess immunomodulatory activity [15]. These findings suggest that AR may have potential for intervention in IBD.

The gut microbiota is essential for intestinal homeostasis and contributes to energy metabolism, nutrient absorption, immune system maturation, and defense against pathogen invasion [16]. Extensive research indicates that the gut microbiota of patients with IBD undergoes marked alterations, characterized by reduced microbial diversity, decreased beneficial bacteria, and expansion of opportunistic pathogens. This dysbiosis is considered a major driver of IBD onset and progression [17]. The intestinal physical barrier, comprising intestinal epithelial cells and intercellular tight junction proteins [18], prevents luminal bacteria, poisons, and other dangerous compounds from entering the bloodstream. Disruption of the intestinal ecosystem compromises epithelial barrier function, allowing lipopolysaccharide (LPS) and other potentially harmful luminal metabolites to translocate into the lamina propria. There, immune recognition triggers inflammatory cascades [19,20], leading to excessive release of proinflammatory cytokines and exacerbating intestinal tissue damage [21,22]. This self-perpetuating cycle—dysbiosis and metabolic disruption causing barrier damage, followed by immune imbalance—represents a key mechanism underpinning persistent inflammation in IBD.

In this context, this study specifically assessed the therapeutic efficacy of AR, in a dextran sulfate sodium (DSS)—caused colitis model in mice. We employed 16S rRNA sequencing and untargeted metabolomics to examine the regulatory effects of AR on the makeup of the microbiota and intestine metabolism in IBD mice, and further evaluated its specific impact on intestinal barrier proteins and pro-inflammatory factor expression. This work aims to establish the novel mechanisms underlying AR’s IBD-alleviating effects, providing experimental evidence for its unique potential as a natural anti-inflammatory agent or an adjunct therapeutic strategy for IBD.

## 2. Results

### 2.1. AR Alleviates Colitis Pathology in IBD Mice

**Figure 1:**
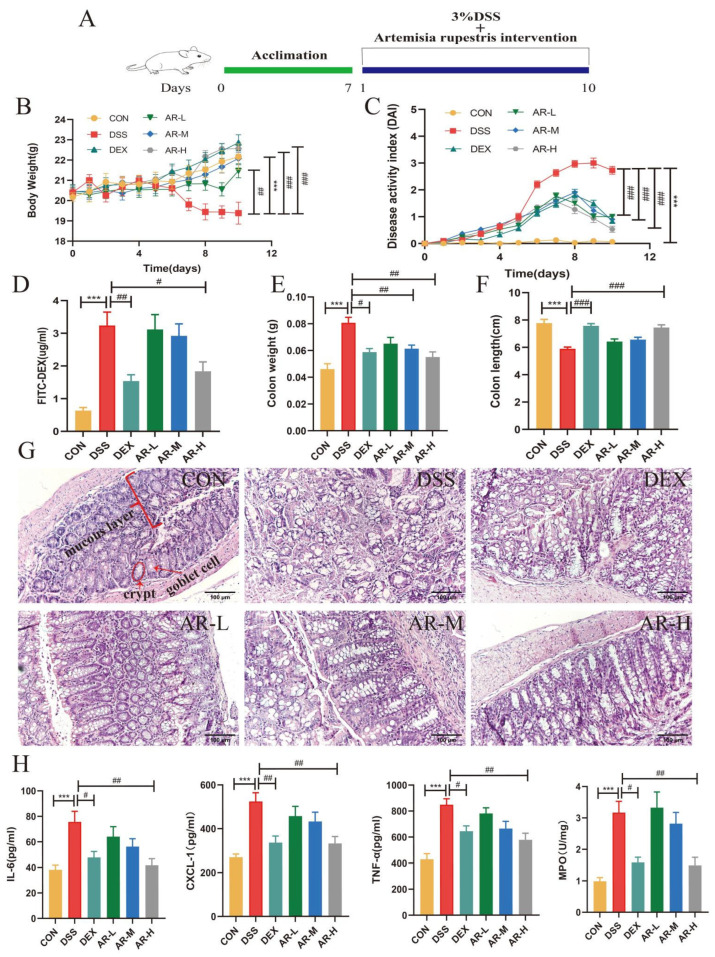
AR alleviates pathological symptoms in mice with DSS-induced colitis. (**A**) Schematic diagram of the experimental design (**B**) Body weight changes (**C**) Disease activity index (DAI) score (**D**) Intestinal permeability assessed by serum FITC-dextran levels (**E**) Colon weight (**F**) Colon length (**G**) Representative hematoxylin and eosin (H&E)-stained colon sections; Scale bar = 100 μm (**H**) Serum concentrations of IL-6, CXCL-1, TNF-α, and MPO. CON, control group; DSS, DSS-induced colitis model group; DEX, dexamethasone group; AR-L, low-dose AR group (25 mg/kg); AR-M, medium-dose AR group (50 mg/kg); AR-H, high-dose AR group (100 mg/kg). For panels (**B**–**F**,**H**), *n* = 10; for panel (**G**), *n* = 6. Compared with the CON group: *** *p* < 0.001. Compared with the DSS group: ^#^ *p* < 0.05, ^##^ *p* < 0.01, ^###^ *p* < 0.001. Data are expressed as the mean ± SEM.

A mouse model of acute colitis caused by DSS was created, as illustrated in Figure 1A. In comparison to the CON group, the DSS group demonstrated characteristic signs of colitis, including substantial weight loss (*p* < 0.05), elevated DAI scores (*p* < 0.01), heightened intestinal permeability (*p* < 0.001), and increased colon weight together with pronounced colon shortening (all *p* < 0.001; Figure 1B–F). Histopathological investigation revealed that DSS administration resulted in significant disruption of colonic mucosal architecture, evidenced by loss of crypt structure, mucosal disarray, pronounced inflammatory cell infiltration, and diminished goblet cell counts (Figure 1G). Simultaneously, serum concentrations of IL-6, CXCL-1, TNF-α, and MPO were markedly increased (*p* < 0.05; Figure 1H). Compared with the DSS group, AR treatment produced dose-dependent improvements, with the AR-H group demonstrating the most pronounced protective effects. AR therapy alleviated the aforementioned pathological changes, partially restoring colonic tissue architecture, reducing inflammatory infiltration, and lowering serum concentrations of pro-inflammatory mediators (*p* < 0.05). Compared with the DEX group, the enhancements in the AR-L and AR-M groups were less significant, whereas the AR-H group showed changes similar to those of the DEX group across many indices. Overall, AR mitigated DSS-induced colonic damage and decreased inflammatory markers in this animal.

### 2.2. AR Restores Intestinal Barrier Stability in IBD Mice

**Figure 2:**
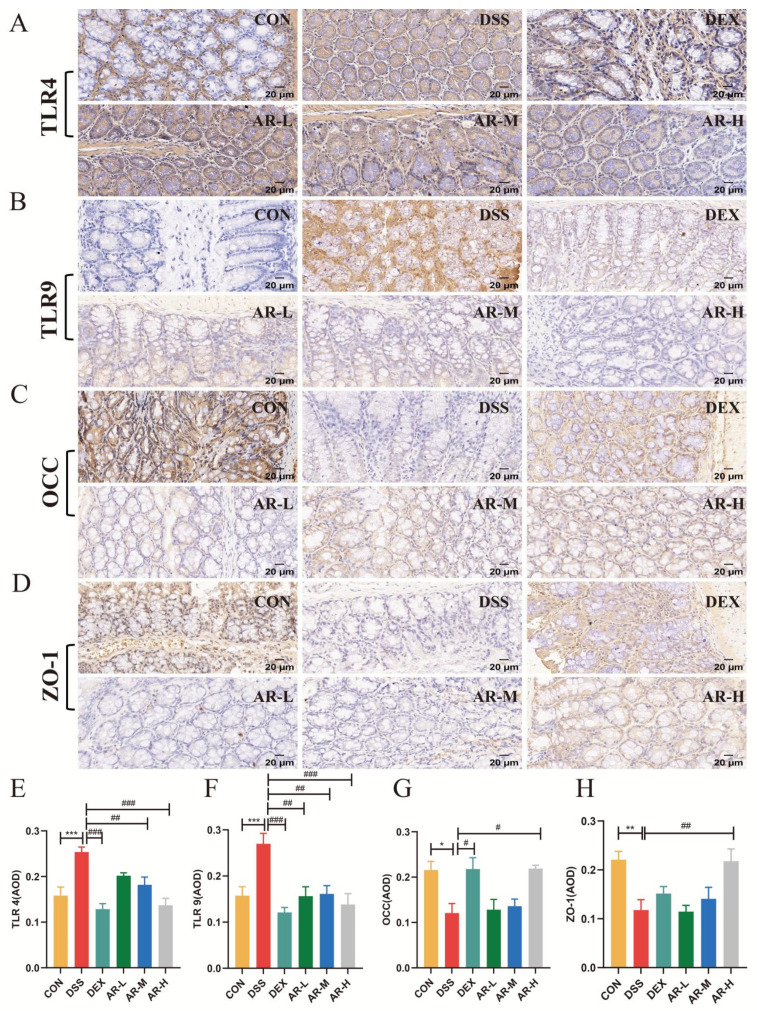
AR improves intestinal barrier function in DSS-induced colitis mice by inhibiting TLR protein expression and restoring tight junction proteins. (**A**–**D**) Representative immunohistochemical staining of TLR4, TLR9, Occludin (OCC), and Zonula Occludens-1 (ZO-1) in colonic tissue, respectively; Scale bar = 20 μm (**E**–**H**) Semi-quantitative analysis of the average optical density (AOD) values for TLR4, TLR9, OCC, and ZO-1, respectively. CON, control group; DSS, DSS-induced colitis model group; DEX, dexamethasone group; AR-L, low-dose AR group (25 mg/kg); AR-M, medium-dose AR group (50 mg/kg); AR-H, high-dose AR group (100 mg/kg). *n* = 6 per group. Compared with the CON group: * *p* < 0.05, ** *p* < 0.01, *** *p* < 0.001. Compared with the DSS group: ^#^ *p* < 0.05, ^##^ *p* < 0.01, ^###^ *p* < 0.001. Data are expressed as the mean ± SEM.

Immunohistochemical labeling was conducted to assess the expression of inflammatory proteins and tight junction proteins in colonic tissue to examine the impact of AR on intestinal barrier function. The findings indicated that, in the DSS group, numerous positive signals for TLR4 and TLR9, along with minimal signals for OCC and ZO-1, were detected in colonic tissue, manifesting as brownish-yellow granules (Figure 2A–D). Quantitative analysis of positive signals in colonic tissue indicated that AR-H and DEX severely diminished TLR4 and TLR9 (*p* < 0.001) levels while significantly elevating OCC and ZO-1 levels in comparison to the DSS group (*p* < 0.05; Figure 2E). The findings demonstrated that AR decreased TLR protein levels while augmenting TJ protein levels; this intervention contributes to the restoration of intestinal barrier integrity and function.

### 2.3. AR Restores Gut Microbiota Diversity and Abundance in IBD Mice

**Figure 3:**
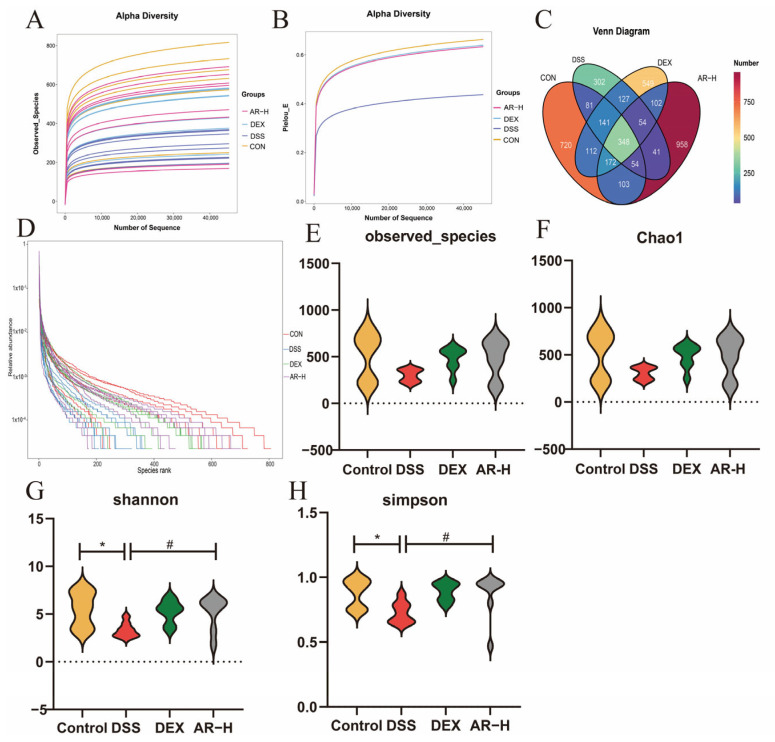
Effects of AR on gut microbiota diversity and richness in IBD mice (*n* = 8). (**A**) Rarefaction curve of the Observed species index. (**B**) Rarefaction curve of Pielou’s Evenness index. (**C**) Venn diagram of amplicon sequence variants (ASVs) among groups. (**D**) Rank-abundance curve of microbial communities. (**E**–**H**) Alpha diversity metrics: Observed species (**E**), Chao1 (**F**), Shannon (**G**), and Simpson (**H**) indices. Compared with the CON group: * *p* < 0.05. Compared with the DSS group: ^#^ *p* < 0.05.

16S rRNA sequencing was conducted to examine the impact of AR on the gut microbiota. Figure 3A,B shows the rarefaction curves based on the number of observed amplicon sequence variants (ASVs; unique sequence variants inferred at single-nucleotide resolution without OTU clustering). The curves for all samples reached a plateau with increasing sequencing depth, indicating that the majority of bacterial taxa in each sample were captured and that the sequencing depth was sufficient for downstream analyses. The Venn diagram illustrates that AR and DEX therapy augmented the total count of ASVs (Figure 3C). AR also mitigated the DSS-induced reduction in richness (Figure 3D). Subsequent diversity analysis revealed a substantial reduction in the Shannon and Simpson indices in the DSS group (all *p* < 0.05), while the Observed Species and Chao1 indices exhibited a declining trend. The Shannon index is sensitive to both community evenness and low-abundance taxa, whereas the Simpson index is more strongly influenced by dominant taxa. In this study, DSS reduced both Shannon and Simpson indices, and AR-H partially restored them. This consistent trend suggests that the DSS-induced decline in alpha diversity was not solely attributable to rare taxa, but was mainly associated with altered community evenness and changes in relatively abundant or dominant taxa. AR partly reinstated these indicators to baseline values (Figure 3E–H). The alterations in ASV count and alpha-diversity indices in the AR-H group were generally analogous to those in the DEX group. These findings suggest that AR partially mitigated DSS-induced alterations in intestinal microbiota diversity and richness in IBD mice.

### 2.4. AR Modulates Key Gut Microbial Taxa in DSS-Induced IBD Mice

**Figure 4:**
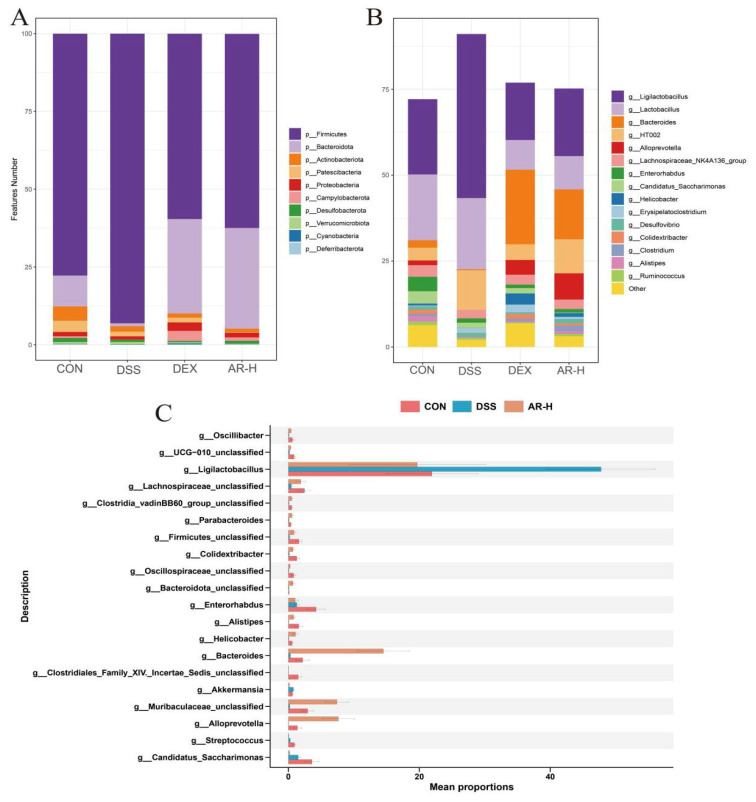
Gut microbiota composition and differential analysis (*n* = 8). (**A**) Bar plot of relative abundance of gut microbiota at the phylum level (**B**) Bar plot of relative abundance of gut microbiota at the genus level (**C**) Significantly different genera among the CON, DSS, and AR-H groups.

Subsequently, we assessed the makeup of the gut microbiota across several taxonomic levels and investigated the regulatory effects of AR on the gut microbiota. At the phylum level, the abundance of *Firmicutes* was higher and the abundance of *Bacteroidota* was lower in the DSS group than in the CON group. After AR-H and DEX intervention, the abundance of *Firmicutes* was lower and the abundance of *Bacteroidota* was higher in the AR-H and DEX groups than in the DSS group (Figure 4A). At the genus level, the abundances of *Ligilactobacillus*, *HT002*, and *Erysipelatoclostridium* increased in the DSS group, whereas the abundances of *Bacteroides*, *Alloprevotella*, and *Lachnospiraceae_NK4A136_group* decreased. However, after AR-H and DEX treatment, the abundances of these taxa returned to control-group levels (Figure 4B). Differential analysis among the three groups showed that the abundance of *Ligilactobacillus*, among others, was significantly increased in the DSS group (*p* < 0.05). Meanwhile, the abundances of *Bacteroides*, *Alloprevotella*, and other genera were significantly decreased (*p* < 0.05). These genera are generally associated with intestinal health, and their reduction indicates a loss of potentially beneficial bacterial communities under DSS induction. AR-H treatment significantly reduced the abundance of *Ligilactobacillus* (*p* < 0.05) and increased the abundances of *Bacteroides* and *Alloprevotella* (*p* < 0.05; Figure 4C). These results indicate that AR treatment attenuated DSS-associated alterations in the bacterial community, including reductions in *Ligilactobacillus* and increases in *Bacteroides* and *Alloprevotella*.

**Figure 5:**
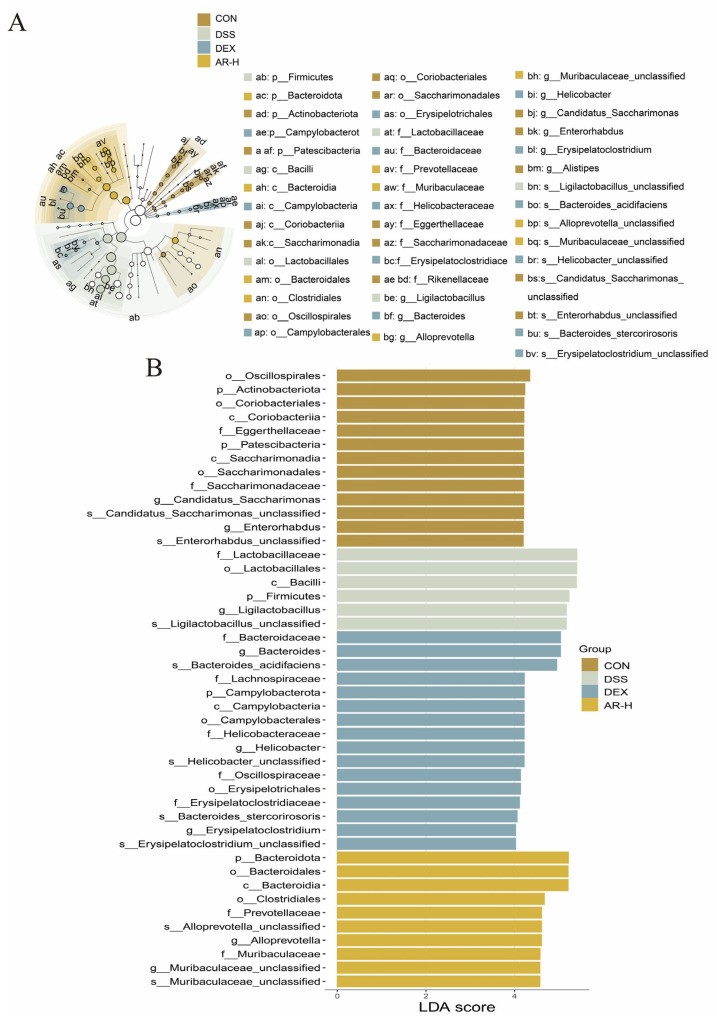
Differential microbial features identified by Linear discriminant analysis effect size (LEfSe) analysis (LDA > 3.0). (**A**) Cladogram. Concentric rings from the center outward represent the taxonomic levels of kingdom, phylum, class, order, family, genus, and species. Each node denotes a taxon at the corresponding level, and node size is proportional to its relative abundance. Uncolored nodes indicate taxa without significant differences among groups, whereas yellow or green nodes indicate significantly different taxa; the color corresponds to the group with higher relative abundance. (**B**) Histogram of LDA scores. Bars represent statistically significant biomarkers; bar color indicates the group in which the taxon is more abundant, and bar length reflects the effect size of the intergroup difference.

Linear discriminant analysis effect size (LEfSe) and linear discriminant analysis (LDA) analysis revealed significant disparities in gut microbiota composition among the groups. The DSS group demonstrated elevated LDA scores for *Ligilactobacillus*, while the AR-H group displayed increased relative abundances of taxa including *Bacteroides* and *Alloprevotella* (*p* < 0.05; Figure 5A).

### 2.5. AR Modulation of Gut Microbiota Correlates with Inflammatory Phenotypes and Barrier Indicators

**Figure 6:**
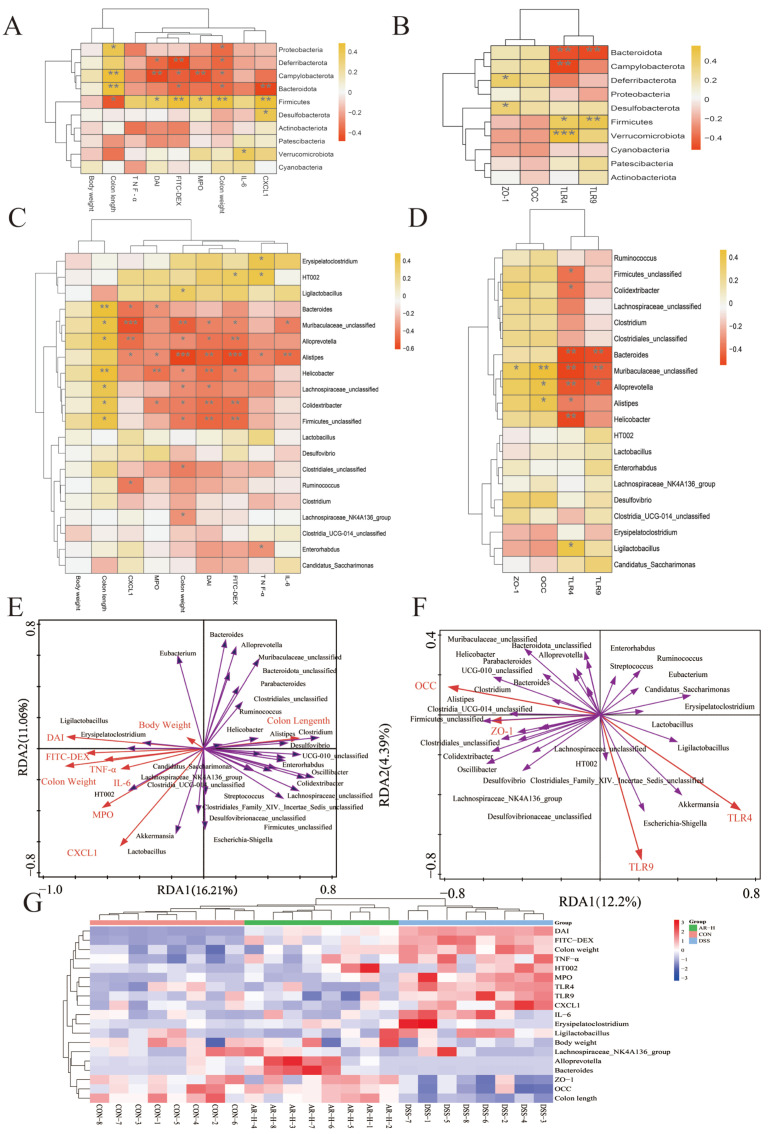
Correlation analysis between the gut microbiota and pathological indices. (**A**) Heatmap of correlations between phylum-level gut microbiota and the assessed indices. (**B**) Heatmap of correlations between phylum-level gut microbiota and the expression of TLR4, TLR9, OCC, and ZO-1. (**C**) Heatmap of correlations between genus-level gut microbiota and the assessed indices. (**D**) Heatmap of correlations between genus-level gut microbiota and the expression of TLR4, TLR9, OCC, and ZO-1. (**E**) Redundancy analysis (RDA) plot of genus-level gut microbiota and the assessed indices. (**F**) RDA plot of genus-level gut microbiota and the expression of TLR4, TLR9, OCC, and ZO-1. (**G**) Heatmap showing correlations between the assessed indices and the relative abundances of *Ligilactobacillus*, *HT002*, *Erysipelatoclostridium*, *Bacteroides*, *Alloprevotella*, and *Lachnospiraceae_NK4A136_group* across samples from the CON, DSS, and AR-H groups. *** *p* < 0.001, ** *p* < 0.01, * *p* < 0.05.

Correlation studies were employed to investigate the relationship between changes in gut microbiota and disease features. Heatmap analyses showed that, at the phylum level, *Firmicutes* was positively correlated with disease activity indices, proinflammatory factors, and TLR proteins, whereas *Bacteroidota* was positively correlated with body weight, colon length, and tight junction proteins (Figure 6A,B). At the genus level, genera including *Bacteroides*, *Alloprevotella*, and *Lachnospiraceae_NK4A136_group* were negatively correlated with inflammatory disease indicators and positively correlated with body weight, colon length, and tight junction proteins. Conversely, genera such as *Ligilactobacillus*, *HT002*, and *Erysipelatoclostridium* were positively correlated with inflammatory disease indicators and negatively correlated with body weight, colon length, and tight junction proteins (Figure 6C,D). Redundancy analysis (RDA) further supported these correlations (Figure 6E,F). Additionally, clustering analyses showed that *Ligilactobacillus*, *HT002*, *Erysipelatoclostridium*, and multiple inflammatory markers clustered with the DSS group, whereas *Bacteroides*, *Alloprevotella*, and *Lachnospiraceae_NK4A136_group*, together with body weight, colon length, and tight junction proteins, clustered with the AR-H group (Figure 6G). Overall, specific microbial taxa were correlated with inflammatory indicators and barrier-related markers across groups.

### 2.6. AR Corrects Metabolic Dysregulation in IBD Mice

**Figure 7:**
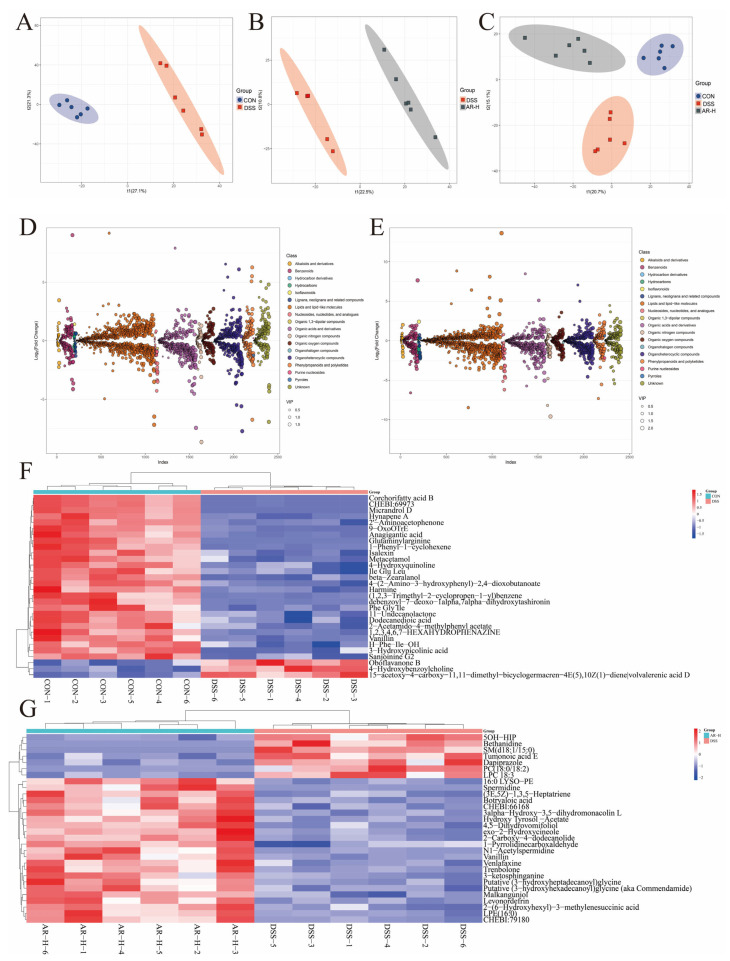
Gut metabolomics analysis (*n* = 6). (**A**) PLS-DA score plot for the CON and DSS groups (**B**) PLS-DA score plot for the DSS and AR-H groups. (**C**) PLS-DA score plot for the CON, DSS, and AR-H groups. (**D**) Volcano plot of differential metabolites between the CON and DSS groups. (**E**) Volcano plot of differential metabolites between the DSS and AR-H groups. (**F**) Clustered heatmap of the top 30 differential metabolites between the CON and DSS groups. (**G**) Clustered heatmap of the top 30 differential metabolites between the DSS and AR-H groups.

To further investigate AR-associated metabolic changes, untargeted metabolomics was used to analyze fecal metabolite profiles across groups. Multivariate statistical analysis (PLS-DA) showed distinct clustering and separation among the CON, DSS, and AR-H groups (Figure 7A–C). The volcano plot analysis showed significant differences in multiple metabolite classes between the DSS group and the CON group. Additionally, compared to the AR-H group, the DSS group also exhibited significant changes in metabolite classes (*p* < 0.05). These results indicate that the DSS group exhibits distinct differences in metabolite distribution and concentration compared to both the CON and AR-H groups (Figure 7D,E). Holistic analysis of clusters was conducted on the 30 most significantly differentially expressed metabolites. The results demonstrated distinct separation among groups and effective clustering within groups. Relative to the CON group, metabolites such as 4-Hydroxybenzoylcholine were positively associated with the DSS group, whereas metabolites such as vanillin were negatively associated. Relative to the DSS group, metabolites including 5OH-HIP and lipid-related molecules such as LPC 18:3 were negatively associated with the AR-H group, whereas metabolites such as vanillin were positively associated with the AR-H group (Figure 7F,G). Overall, DSS-associated metabolic shifts were consistent across samples, and the AR-H group showed an overall reversal trend in fecal metabolic profiles relative to the DSS group.

**Figure 8:**
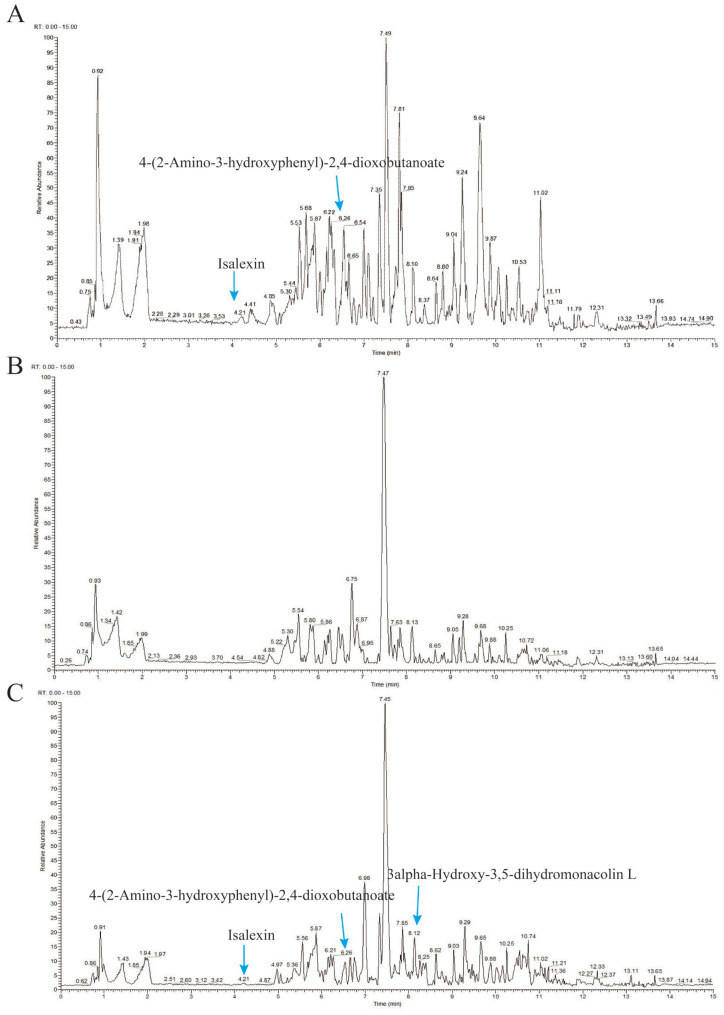
Representative UPLC-ESI-MS/MS chromatograms of fecal metabolites acquired in negative-ion mode. (**A**) CON group; (**B**) DSS group; (**C**) AR-H group. Metabolite annotations were added only when the corresponding peaks could be confidently assigned based on retention time, precursor ion information, and MS/MS annotation. In negative-ion mode, 4-(2-amino-3-hydroxyphenyl)-2,4-dioxobutanoate, Isalexin, and 3alpha-hydroxy-3,5-dihydromonacolin L were annotated in the corresponding chromatograms. In the representative DSS chromatogram, these metabolites were not labeled because no clearly resolved peaks suitable for confident visual annotation were observed at the expected retention-time regions. This may be associated with weak signal intensity, limited signal-to-noise ratio, ionization efficiency, matrix effects, or peak overlap; however, the exact reason cannot be determined from the representative chromatogram alone. Therefore, the absence of labels in panel B should not be interpreted as definitive absence of these metabolites.

**Figure 9:**
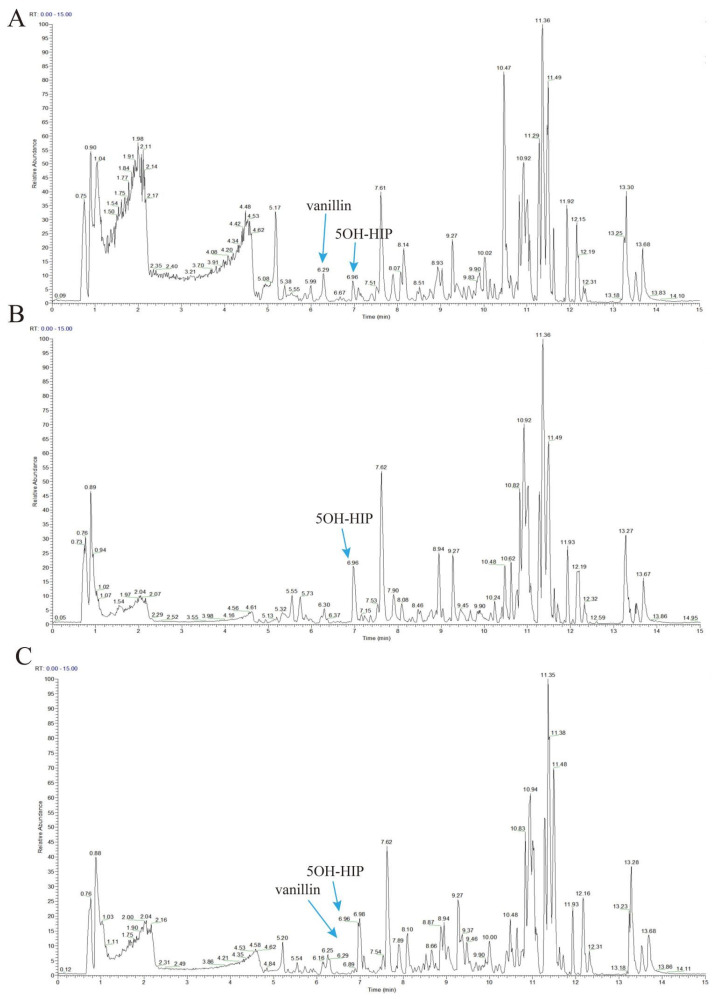
Representative UPLC-ESI-MS/MS chromatograms of fecal metabolites acquired in positive-ion mode. (**A**) CON group; (**B**) DSS group; (**C**) AR-H group. Metabolite annotations were added only when the corresponding peaks could be confidently assigned based on retention time, precursor ion information, and MS/MS annotation. In positive-ion mode, vanillin and 5OH-HIP were annotated in the corresponding chromatograms. 5OH-HIP showed visually identifiable signals in the representative chromatograms of all three groups, whereas vanillin was annotated only in panels with clearly resolved signals. In the representative DSS chromatogram, vanillin was not labeled because its signal at the expected retention-time region was not sufficiently resolved for confident visual annotation. This may reflect weak signal intensity, limited signal-to-noise ratio, matrix effects, ionization-related variability, or local peak interference.

**Figure 10:**
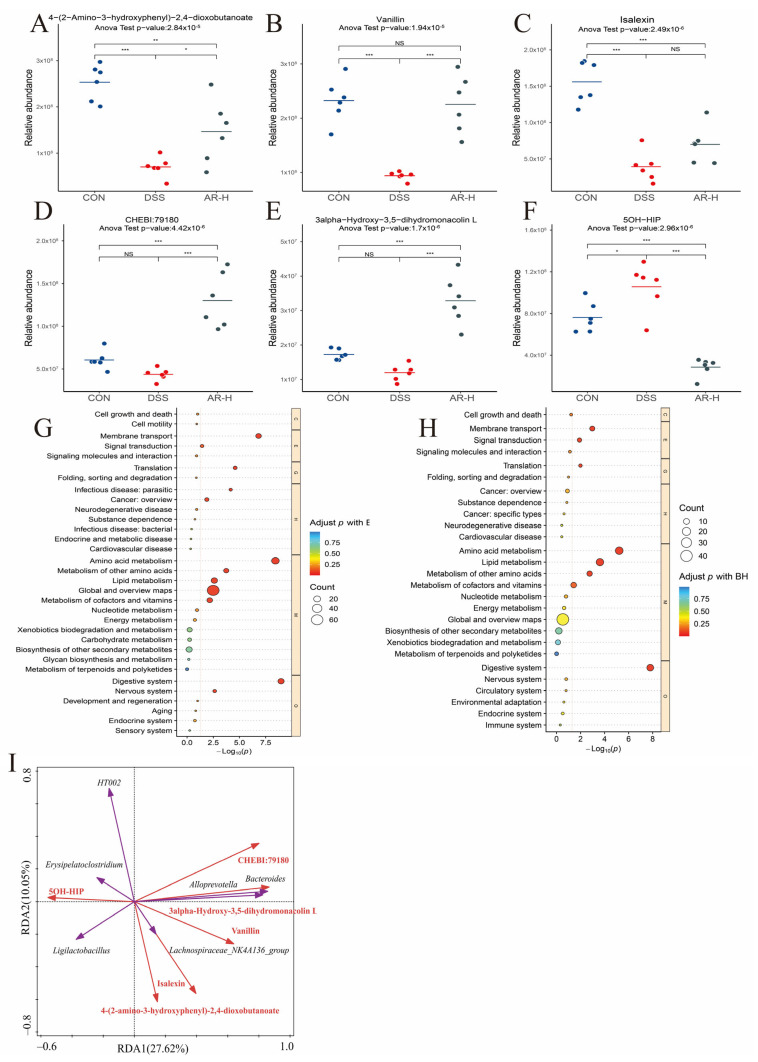
Shared differential metabolites and RDA-based correlation analysis. (**A**–**F**) Scatter plots of shared differential metabolites among the CON, DSS, and AR-H groups. (**G**) KEGG pathway enrichment analysis of differential metabolites between the CON and DSS groups. (**H**) KEGG pathway enrichment analysis of differential metabolites between the DSS and AR-H groups. (**I**) RDA plot showing the associations between shared differential metabolites and the genera *Ligilactobacillus*, *HT002*, *Erysipelatoclostridium*, *Bacteroides*, *Alloprevotella*, and *Lachnospiraceae_NK4A136_group*. *** *p* < 0.001, ** *p* < 0.01, * *p* < 0.05.

UPLC-ESI-MS/MS results further showed clear differences in metabolic profiles among the CON, DSS, and AR-H groups (Figure 8A–C and Figure 9A–C). Through the intersection of differential metabolites identified in the CON vs. DSS and DSS vs. AR-H comparisons, six common differential metabolites were discerned: 4-(2-Amino-3-hydroxyphenyl)-2,4-dioxobutanoate, vanillin, CHEBI:79180 ((2E)-19-hydroxynonadec-2-enoic acid), 5OH-HIP, Isalexin, and 3alpha-Hydroxy-3,5-dihydromonacolin L (Table 1). Abundance analysis showed that 5OH-HIP was significantly increased in the DSS group (*p* < 0.05), while 4-(2-Amino-3-hydroxyphenyl)-2,4-dioxobutanoate, vanillin, and Isalexin were dramatically diminished (all *p* < 0.001). CHEBI:79180 and 3alpha-Hydroxy-3,5-dihydromonacolin L exhibited a declining trend. The AR-H treatment markedly ameliorated the DSS-induced alterations (*p* < 0.05; Figure 10A–F). Pathway analysis revealed that, in the CON vs. DSS comparison, differentially expressed metabolites were primarily enriched in pathways such as Global and overview maps and Amino acid metabolism. In the DSS vs. AR-H comparison, enrichment was mainly observed in pathways including Lipid metabolism and Digestive system (Figure 10G,H). Additionally, RDA analysis revealed correlations between gut microbiota and significant differential metabolites: *Ligilactobacillus*, *HT002*, and *Erysipelatoclostridium* exhibited positive correlations with 5OH-HIP, while *Bacteroides*, *Alloprevotella* and *Lachnospiraceae_NK4A136_group* demonstrated positive correlations with 4-(2-Amino-3-hydroxyphenyl)-2,4-dioxobutanoate, vanillin, Isalexin, CHEBI:79180, and 3alpha-Hydroxy-3,5-dihydromonacolin L (Figure 10I, Table 2). Taken together, AR intervention was associated with concordant correlation changes in intestinal metabolic regulation, key metabolite levels, and specific bacterial populations, consistent with an improvement in metabolic disorders associated with DSS-induced colitis.

## 3. Discussion

**Figure 11:**
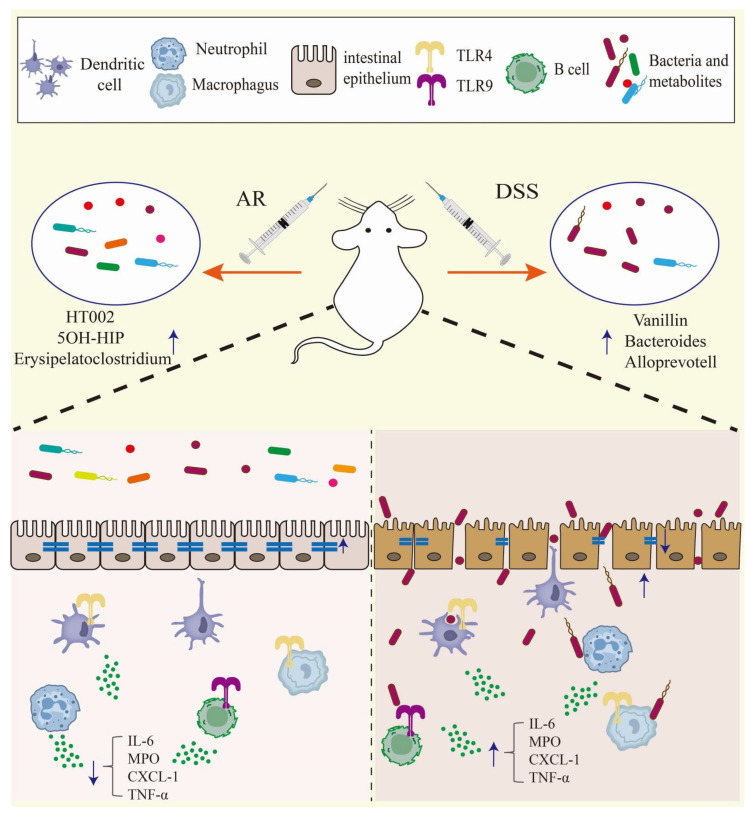
Proposed mechanism of AR in DSS-induced colitis. DSS treatment induces changes in the intestinal microbiota and metabolite profiles, leading to disruption of epithelial tight junctions and increased permeability. This facilitates contact between bacteria/metabolites and epithelial and immune cells, accompanied by increased expression of TLR4 and TLR9, which promotes the release of inflammatory factors and contributes to immune dysregulation and inflammatory responses, including elevated levels of IL-6, MPO, CXCL-1, and TNF-α, thereby initiating and amplifying an inflammatory cycle. AR intervention helps to reshape the microbiota-metabolite microenvironment, reduce TLR4 and TLR9 expression and inflammatory factor levels, and promote recovery of barrier structure, thereby interrupting the inflammatory cycle and maintaining intestinal homeostasis. Note: In the schematic, Upward arrows (↑) indicate an increase or up-regulation, while downward arrows (↓) indicate a decrease or down-regulation.

This study found that AR attenuates DSS-induced gut microbiota imbalance, reduces microbiota-associated inflammatory stimulation, restores selected beneficial metabolites and metabolic pathways, inhibits TLR4- and TLR9-associated inflammatory signaling, and promotes recovery of tight junction protein expression, thereby decreasing intestinal permeability and inflammatory responses and ultimately alleviating IBD symptoms (Figure 11).

The DSS-induced colitis model has been widely used in IBD research because it is simple, reproducible, and recapitulates several key features of human ulcerative colitis, including epithelial injury, barrier disruption, inflammatory cell infiltration, colon shortening, diarrhea, and bloody stool. However, DSS primarily induces chemical epithelial damage and acute mucosal inflammation; therefore, it does not fully reproduce the genetic susceptibility, chronic relapsing course, and complex immune regulation of human IBD. In the present study, DSS treatment induced marked gut microbial dysbiosis and fecal metabolic disturbance. The overall microbial pattern was broadly consistent with previous DSS colitis studies, including reduced microbial diversity, expansion of inflammation-associated taxa, and depletion of potentially beneficial or SCFA-related bacteria [23]. Specifically, DSS increased the relative abundance of *Ligilactobacillus*, *HT002*, and *Erysipelatoclostridium*, while reducing *Bacteroides*, *Alloprevotella*, and *Lachnospiraceae_NK4A136_group*. Although the overall direction of dysbiosis was consistent with previous reports, differences in specific taxa may be explained by DSS concentration, exposure duration, mouse strain, housing conditions, baseline microbiota, sample type, sequencing region, and bioinformatic pipeline [24,25]. At the metabolic level, DSS caused broad alterations in fecal metabolites, mainly involving amino acid and lipid metabolism, which is also consistent with previous reports in DSS colitis models and human IBD. In our study, DSS increased 5OH-HIP and decreased 4-(2-amino-3-hydroxyphenyl)-2,4-dioxobutanoate, vanillin, Isalexin, CHEBI:79180, and 3alpha-hydroxy-3,5-dihydromonacolin L, whereas AR-H partially reversed these changes. Thus, the robustness of our findings is supported not by a single microbial taxon or metabolite, but by the concordant changes in gut microbiota, fecal metabolites, inflammatory markers, TLR protein, and intestinal barrier indicators.

The stability of the gut microbial community is essential for maintaining intestinal homeostasis. Dysbiosis and reduced microbial diversity are common pathological features of IBD. Dysbiosis typically manifests as an overgrowth of pro-inflammatory bacteria that inappropriately activate immune responses, while a reduction in beneficial bacteria further exacerbates intestinal inflammation [26,27,28]. Consistent with these features, our study identified marked alterations in the microbial community in DSS-induced IBD mouse models. DSS-induced colitis has been widely used to study gut microbiota dysbiosis; therefore, our 16S rRNA sequencing results should be interpreted in the context of previous DSS model studies rather than as a first description of this phenomenon. Overall, our DSS-versus-CON results are consistent with previous reports showing reduced microbial diversity, expansion of inflammation-associated or facultative anaerobic taxa, and depletion of bacteria associated with short-chain fatty acid production. In our study, DSS increased the relative abundances of *Ligilactobacillus*, *HT002*, and *Erysipelatoclostridium*, while reducing *Bacteroides*, *Alloprevotella*, and *Lachnospiraceae_NK4A136_group*. Differences among studies may be explained by variations in DSS concentration, exposure duration, mouse strain, housing conditions, baseline microbiota, sample type, sequencing region, and bioinformatic pipeline. Thus, the robustness of our findings is supported by the concordance among microbial diversity, taxonomic shifts, inflammatory phenotypes, barrier protein expression, and metabolomic alterations, rather than by a single taxonomic change. The DSS group exhibited heightened relative abundances of *Ligilactobacillus*, *HT002*, and *Erysipelatoclostridium*. Correlation analysis further showed that these taxa were positively correlated with pathological indicators, including increased DAI, TLR4, and TLR9 protein expression, and proinflammatory factor levels (IL-6, CXCL-1, TNF-α, and MPO). *Ligilactobacillus* is typically regarded as a health-promoting lactic acid bacterium and a probiotic genus. Prior research has indicated that specific strains can diminish inflammation and mitigate tissue damage in animal models of colitis; thus, an elevated presence is frequently seen as a potentially advantageous indicator in many circumstances [29,30]. However, in the context of acute mucosal injury and inflammatory stress observed in this study, this finding may have different biological implications and warrants further consideration. In IBD, intestinal epithelial injury and inflammatory responses are often accompanied by alterations in the local redox environment, including oxidative stress and increased luminal oxygen tension. These changes can confer growth advantages to aerotolerant or facultative anaerobic microbiota and may contribute to inflammation-associated dysbiosis [31,32]. Acute DSS-induced colitis has been reported to alter the intestinal oxygen environment [33]. In addition, *Ligilactobacillus* comprises facultative anaerobic, oxygen-tolerant fermentative bacteria [34]. Therefore, the enrichment of *Ligilactobacillus* in the DSS group may reflect adaptive growth under an oxidative intestinal environment, consistent with dysbiosis rather than a simple proliferation of beneficial bacteria. At present, the physiological functions of *HT002* remain poorly characterized. However, its positive correlations with multiple inflammatory markers suggest that it may function as an opportunistic pathogen or an inflammation-associated microorganism. Altered intestinal microenvironments can confer growth advantages to specific bacteria; thus, *HT002* amplification may similarly represent an adaptive response to pathological conditions and may contribute to or exacerbate chronic inflammation. *Erysipelatoclostridium* has been reported in multiple clinical studies to be more abundant in patients with IBD [35,36]. Moreover, some representatives of this genus have demonstrated the ability to attach to the intestinal epithelium and elicit Th17 responses [37], indicating that their proliferation may correlate with DSS-induced intensification of intestinal inflammation. Dysbiosis and its related microbial constituents can traverse mucosal barriers and be detected by pattern recognition receptors, including TLR4 and TLR9. This identification can initiate inflammatory pathways, such as MyD88/NF-κB, resulting in the production of proinflammatory mediators [38]. Furthermore, TLR9 expression has been reported to correlate positively with inflammatory activity and proinflammatory factors such as IL-6 and TNF-α [39]. This is consistent with the observed upregulation of TLR4 and TLR9 in the DSS group, together with elevated IL-6, CXCL-1, TNF-α, and MPO, supporting a role for TLR-associated inflammatory responses in DSS-induced disease progression.

Conversely, the relative abundances of *Bacteroides*, *Alloprevotella*, and *Lachnospiraceae_NK4A136_group* decreased in the DSS group and were negatively correlated with pathological indicators. These genera are associated with the production of short-chain fatty acids (SCFAs). Specifically, *Bacteroides* and *Alloprevotella* can produce SCFAs such as acetate, which acts as a ligand for GPR43 and thereby suppresses inflammatory responses [40,41]. *Lachnospiraceae_NK4A136_group* is a butyrate-producing group, and butyrate plays a crucial role in maintaining mucosal homeostasis and barrier integrity. Following AR treatment, the abnormal increases in *Ligilactobacillus*, *HT002*, and *Erysipelatoclostridium* were suppressed, and the relative abundances of *Bacteroides*, *Alloprevotella*, and *Lachnospiraceae_NK4A136_group* increased, in parallel with improvements in inflammatory phenotypes. In summary, AR may suppress DSS-induced intestinal inflammation by limiting the expansion of inflammation-associated taxa and promoting recovery of taxa associated with SCFA production, thereby reducing microbiota-associated stimulation and TLR overactivation after barrier injury and ultimately decreasing proinflammatory factor expression.

Tight junction proteins (TJs) are essential for maintaining intestinal barrier integrity and regulating permeability. ZO-1 and OCC are key components of tight junctions. This study found that DSS-induced colitis was associated with reduced OCC and ZO-1 expression in colonic tissue, along with increased intestinal permeability. A correlation study indicated that OCC and ZO-1 expression exhibited a negative correlation with *Ligilactobacillus*, *HT002*, and *Erysipelatoclostridium*, while demonstrating a positive correlation with *Bacteroides*, *Alloprevotella*, and *Lachnospiraceae_NK4A136_group*. Post-AR treatment, OCC and ZO-1 expression levels were analogous to those in the CON group, and intestinal permeability was enhanced, signifying a protective effect on barrier integrity. Previous studies have indicated that *Ligilactobacillus* enrichment likely reflects adaptation to the pathological microenvironment. Conversely, *HT002*’s positive correlation with decreased barrier protein expression suggests potential involvement in impaired barrier stability. *Erysipelatoclostridium* has been reported to show an upward trend in clinical IBD studies, suggesting that its overgrowth may indirectly inhibit TJ protein recovery and exacerbate barrier damage by enhancing inflammatory stimulation or altering the metabolic environment. In contrast, the populations of bacterial genera including *Bacteroides*, *Alloprevotella*, and *Lachnospiraceae_NK4A136_group* diminished, which may decrease the availability of intestinal short-chain fatty acids (SCFAs), potentially hindering the restoration of tight junction (TJ) proteins and compromising SCFA-mediated protection of the intestinal barrier [42]. Therefore, AR-associated recovery of these SCFA-producing microbial communities may represent a key ecological basis for barrier repair.

DSS-induced barrier disruption can facilitate translocation of bacterial components into the lamina propria, thereby activating pattern recognition receptors such as TLRs and amplifying inflammatory signals, including IL-6, CXCL-1, TNF-α, and MPO. These proinflammatory factors can further damage TJ structures, creating a vicious cycle of inflammation and barrier impairment [43]. Previous studies have reported that TNF-α increases permeability by activating MLCK, leading to TJ protein endocytosis [44]. In addition, reactive oxygen species (ROS) generated by MPO, such as HOCl, can cause oxidative protein modifications and impair barrier stability [45]. Moreover, CXCL-1 can recruit neutrophils to inflammatory sites, amplifying local inflammation and exacerbating epithelial damage, thereby indirectly contributing to TJ protein loss. Collectively, these factors contribute to reduced OCC and ZO-1 expression, along with increased permeability. AR therapy may interrupt this vicious cycle by upregulating OCC and ZO-1 expression, reducing intestinal permeability, and promoting restoration of gut microbiota associated with barrier function, thereby improving barrier stability.

Intestinal metabolites are key mediators of intestinal immune homeostasis and barrier integrity. In the DSS group, numerous metabolites showed significant changes, indicating disrupted intestinal metabolic homeostasis under IBD conditions. After AR-H intervention, the overall metabolite profile showed recovery and shifted toward that of the CON group. These findings suggest that AR partially reverses DSS-induced metabolic dysregulation overall. Concentrating on six distinct, differentially expressed metabolites, 5OH-HIP was markedly elevated in the DSS group and had a positive correlation with *Ligilactobacillus*, *HT002*, and *Erysipelatoclostridium*. Prior research indicates that 5OH-HIP serves as an intermediate metabolite in bacterial cholesterol breakdown processes [46,47]. Thus, the abnormal accumulation of 5OH-HIP in the DSS group may reflect impaired cholesterol degradation by the gut microbiota, resulting in elevated 5OH-HIP levels. This alteration may further disrupt the intestinal microenvironment and undermine gut homeostasis. Following AR intervention, 5OH-HIP levels significantly decreased, suggesting that AR may reduce 5OH-HIP toward baseline by regulating the microbiota and its metabolic functions, thereby contributing to the recovery of intestinal microenvironment stability. In contrast, in the DSS group, the abundances of 4-(2-Amino-3-hydroxyphenyl)-2,4-dioxobutanoate, Vanillin, Isalexin, CHEBI:79180, and 3alpha-Hydroxy-3,5-dihydromonacolin L were significantly decreased and were positively correlated with SCFA-producing taxa including *Bacteroides*, *Alloprevotella*, and *Lachnospiraceae_NK4A136_group*. Levels of these metabolites increased following AR intervention. Existing literature provides limited information on the physiological function of 4-(2-amino-3-hydroxyphenyl)-2,4-dioxobutanoate. Its structure contains a 2-amino-3-hydroxyphenyl moiety and an oxo-butanoate/dicarbonyl side chain, which are partially similar to structural features of kynurenine-related metabolites, particularly hydroxylated aromatic intermediates derived from tryptophan metabolism. Therefore, we cautiously suggest that this metabolite may be associated with kynurenine-related metabolism; however, this inference is based on structural annotation and requires further validation by targeted metabolomics or isotope-tracing experiments [48]. The kynurenine pathway is frequently associated with immune tolerance, epithelial energy metabolism, and AhR signaling in IBD. The reduction in this metabolite may indicate impaired tryptophan-kynurenine metabolism, potentially contributing to exacerbated inflammation. Vanillin is a natural phenolic compound with antioxidant and anti-inflammatory activities. Studies indicate that it suppresses MAPK and NF-κB signaling, thereby exerting anti-inflammatory effects in IBD models [49]. Thus, decreased vanillin levels may reflect depletion or reduced production of antioxidant or anti-inflammatory metabolites within the intestinal lumen, thereby weakening the gut’s capacity to resist inflammatory injury. Isalexin is a plant antitoxin from cruciferous plants that can undergo fungal biotransformation and may represent an exogenous metabolite derived from plant sources or gut microbial conversion. Its homolog, arvelexin, has been shown to inhibit NF-κB, alleviate IBD, and reduce inflammatory markers [50], suggesting that decreased Isalexin levels may reflect perturbations in exogenous indole metabolites, although the specific mechanism remains to be further validated. CHEBI:79180 belongs to the class of ω-hydroxy monounsaturated long-chain fatty acids [51]. Previous studies have shown that the gut microbiota can produce bioactive hydroxy fatty acids, and that some of these, acting as PPARγ agonists, can improve DSS-induced colitis [52]. Therefore, decreased CHEBI:79180 levels in the DSS group suggest that protective lipid mediators may be depleted, thereby promoting a persistent inflammatory state. At present, the physiological significance of 3α-Hydroxy-3,5-dihydromonacolin L in the intestine remains unclear. Its reduction in the DSS group may indicate altered microbial secondary metabolism or lipid homeostasis under IBD conditions. Further studies are needed to clarify its origin and function. Although bile acid metabolism is closely linked to the gut microbiota and intestinal inflammation, bile acid-related metabolites were not among the top differential metabolites shown in Figure 7. Because this study used untargeted metabolomics to characterize global fecal metabolic changes rather than targeted bile acid profiling, we avoided overinterpreting bile acid-related signals without targeted quantification and authentic-standard validation. Future targeted studies are needed to clarify the role of the bile acid-gut microbiota axis in the protective effects of AR against DSS-induced colitis.

At the pathway level, in the CON vs. DSS comparison, differentially expressed metabolites were primarily enriched in pathways such as amino acid metabolism. Previous studies have indicated that, in enteritis, pathways related to amino acid metabolism and oxidative stress are frequently dysregulated. Amino acids not only influence epithelial energy supply and protein synthesis but also participate in the regulation of mucosal inflammation by generating various immunologically active small molecules [53]. In the DSS vs. AR-H comparison, differentially expressed metabolites were mainly enriched in pathways such as lipid metabolism. Impaired lipid metabolism may elevate the synthesis of pro-inflammatory lipid mediators while diminishing the generation of anti-inflammatory lipids. These imbalanced lipid molecules can directly participate in inflammatory responses and immune cell activation, thereby amplifying the inflammatory cascade. The observed decrease in CHEBI:79180 in the DSS group, followed by its recovery after AR-H treatment, further supports a pro-inflammatory bias in lipid metabolism pathways, with AR partially reversing this trend. In summary, the DSS group exhibited enhanced pro-inflammatory signaling and a relative deficiency in protective and anti-inflammatory metabolites, accompanied by imbalances in key pathways, including amino acid and lipid metabolism. Collectively, these factors may create a metabolic environment conducive to sustained inflammation and intestinal damage. By correcting disruptions in key metabolites and metabolic pathways, AR may act in concert with the gut microbiota to suppress inflammation and promote barrier repair.

This study has several limitations. First, the AR water extract used in this study was not subjected to systematic phytochemical characterization. Therefore, although previous studies have reported multiple constituents in *Artemisia rupestris*, the specific chemical profile and active constituents of the extract used in the present experiment remain to be further verified by UPLC/HPLC fingerprinting, LC-MS/MS identification, and authentic-standard validation. Second, the DSS-induced acute colitis model, while reflecting mucosal injury and amplified inflammation, differs from the chronic, relapsing course of IBD. Accordingly, the long-term efficacy of AR in chronic IBD requires further validation. Third, correlation analyses indicate associations between variables but do not establish causality. Future studies could integrate metagenomic analyses, fecal microbiota transplantation (FMT), and targeted strain supplementation to validate the roles of key taxa in mediating AR-associated effects. Finally, although this study focused on changes in TLR and TJ proteins, further validation of downstream signaling pathways is needed to establish a more comprehensive mechanistic framework.

## 4. Materials and Methods

### 4.1. Materials

Zonula occludens-1 (ZO-1) rabbit polyclonal antibody (pAb; catalog No. 61-7300) and Occludin (OCC) rabbit pAb (catalog No. 71-1500) were obtained from Thermo Fisher Scientific (Waltham, MA, USA); Toll-like receptor 4 (TLR4) rabbit pAb (catalog No. NB100-56581) was obtained from Novus Biologicals (Centennial, CO, USA); and Toll-like receptor 9 (TLR9) rabbit pAb (catalog No. bs-2717R) was obtained from Bioss Antibodies (Beijing, China). Dextran sulfate sodium salt (DSS; CAS No. 9011-18-1; average molecular weight, 40 kDa; lot No. c12750877) was purchased from Shanghai Kelin Biochemical Technology Co., Ltd. (Shanghai, China). Dexamethasone injection was purchased from Shanghai Modern Hasen (Shangqiu) Pharmaceutical Co., Ltd. (Shangqiu, China), batch No. 1905190231. Fecal Occult Blood (FOB) test kit (Shanghai Enzyme-Linked Biotechnology Co., Ltd., Shanghai, China; batch No. 0701A22).

### 4.2. Preparation of the Aqueous Extract of Artemisia rupestris

AR was purchased from Yili Mingzhu Technology Development Co., Ltd. (Urumqi, Xinjiang, China). The dried whole herb was pulverized and extracted with purified water at a material-to-solvent ratio of 1:10 (*w*/*v*). After soaking at room temperature for 30 min, the mixture was reflux-extracted three times. The extracts were combined, filtered, and concentrated under reduced pressure to obtain the dried aqueous extract. Before administration, the dried extract was freshly dissolved in distilled water and diluted to the required working concentrations. Based on previous phytochemical reports, flavonoids and sesquiterpenoids are considered major bioactive constituents of AR.

### 4.3. Experimental Animals

Male C57BL/6J mice, aged 6 to 8 weeks and weighing 20 to 24 g, were procured from the Experimental Animal Center of Xinjiang Medical University.

### 4.4. Establishment of IBD Mouse Model and Drug Intervention

After 7 days of acclimatization, mice were randomly divided into six groups using a random-number method: normal control group (CON), DSS model group (DSS), dexamethasone group (DEX, 0.4 mg/kg), low-dose AR group (AR-L, 25 mg/kg), medium-dose AR group (AR-M, 50 mg/kg), and high-dose AR group (AR-H, 100 mg/kg), with 10 mice in each group. Acute colitis was induced by administering 3% DSS in sterilized drinking water for 10 consecutive days. DSS was freshly dissolved in sterilized drinking water at a final concentration of 3% (*w*/*v*) immediately before use and supplied ad libitum to mice in the DSS, DEX, and AR-treated groups during the modeling period. The DSS-containing drinking water was replaced every two days to maintain solution stability and consistent exposure. Mice in the CON group received sterilized drinking water without DSS. During the modeling period, AR was administered once daily by oral gavage at doses of 25, 50, or 100 mg/kg, corresponding to the AR-L, AR-M, and AR-H groups, respectively. The gavage volume was 10 mL/kg. Mice in the DSS group received an equal volume of sterilized water by oral gavage. Mice in the DEX group received dexamethasone at 0.4 mg/kg by intraperitoneal injection once daily. The AR doses were selected based on preliminary dose-ranging observations showing that 25–100 mg/kg AR was well tolerated and allowed evaluation of dose-dependent effects in DSS-induced colitis. Body weight was recorded daily throughout the experimental period, and the disease activity index (DAI) was evaluated daily to monitor the progression of colitis.

The disease activity index (DAI) was assessed based on body weight loss, stool consistency, and fecal bleeding/occult blood. Body weight loss was scored as follows: 0, no weight loss; 1, 1–5% weight loss; 2, 6–10% weight loss; 3, 11–15% weight loss; and 4, more than 15% weight loss. Stool consistency was scored as follows: 0, normal stool; 2, watery diarrhea; and 4, severe watery diarrhea with blood. Fecal Occult Blood (FOB) test kit (Shanghai Enzyme-Linked Biotechnology Co., Ltd., Shanghai, China; batch No. 0701A22) and scored as follows: 0, no blood; 2, presence of blood; and 4, gross bleeding. The final DAI score was calculated as the average of the scores for body weight loss, stool consistency, and fecal bleeding/occult blood.

On day 11, mice were anesthetized and euthanized with sodium pentobarbital (100–150 mg/kg). The entire colon was carefully excised from the ileocecal junction to the anal verge. Fecal contents were gently removed by rinsing with cold PBS, and the colon was placed flat on a moist surface without stretching. Colon length was measured in centimeters using a metric ruler from the ileocecal junction to the anal verge. All measurements were performed by an investigator blinded to the group allocation.

### 4.5. Intestinal Permeability Assessment

FITC-dextran was used as a tracer to assess intestinal mucosal permeability. After a 4 h fast, mice in each group were orally administered fluorescein isothiocyanate-dextran (FITC-dextran; 800 mg/kg) by gavage. Four hours after gavage, blood was collected from the retro-orbital venous plexus. The blood samples were centrifuged at 2000 rpm for 20 min at 4 °C to separate the serum. The serum was transferred and stored at −80 °C, and repeated freeze–thaw cycles were avoided. FITC-dextran levels in serum were measured at an excitation wavelength of 485 nm and an emission wavelength of 535 nm. Intestinal mucosal permeability was evaluated based on the serum FITC-dextran level.

### 4.6. Hematoxylin and Eosin Staining

Colon tissues were fixed in 4% paraformaldehyde for 24 h, gradually dehydrated through an ethanol gradient, cleared in xylene, and embedded in paraffin. Serial sections (5 μm thick) were cut using a rotary microtome and mounted on slides. After deparaffinization in xylene and rehydration through a descending ethanol series, sections were stained with hematoxylin for 5 min, differentiated in 1% hydrochloric acid–ethanol for several seconds, and blued in running tap water. Thereafter, sections were counterstained with eosin for 2 min. Following staining, the sections were dehydrated through graded ethanols, cleared in xylene, and permanently mounted with neutral balsam. Histological images were acquired using a Nikon Eclipse Ni-U light microscope (Nikon, Tokyo, Japan) under identical imaging conditions. The magnification and scale bars were kept consistent across groups, and image panels were assembled at high resolution to ensure readability.

### 4.7. ELISA Analysis

Blood specimens were collected, and serum was extracted via centrifugation and preserved at −80 °C until analysis. Serum samples were used for the measurement of pro-inflammatory cytokines and for the assessment of intestinal permeability. The concentrations of IL-6, CXCL-1, and TNF-α in mouse serum were measured using commercial ELISA kits following the manufacturer’s instructions. Standards and samples were introduced to the pre-coated plates, followed by sequential incubation with the detection antibody and enzyme-conjugated secondary antibody, culminating in development with TMB substrate, as delineated by the manufacturer. Absorbance was recorded at 450 nm, and concentrations were derived from the standard curve. Notably, blood cultures for bacterial translocation were not performed in this study.

### 4.8. Immunohistochemistry

Paraffin-embedded colorectal tissue sections were removed from paraffin and hydrated again, and thereafter underwent heat-induced antigen retrieval to restore antigen epitopes. Endogenous peroxidase activity was inhibited using hydrogen peroxide as a solvent, and non-specific binding sites were obstructed with sera. Sections were incubated overnight at 4 °C with primary antibodies against TLR4 (2.5 μg/mL), TLR9 (1:200), OCC (2.5 μg/mL), and ZO-1 (2.5 μg/mL). After washing with PBS, sections were incubated with HRP-conjugated goat anti-rabbit IgG secondary antibody (1:500) for 1 h at room temperature. Target proteins were seen with a DAB chromogenic detection technique. Nuclei were counterstained with hematoxylin, subsequently subjected to dehydration, clearing, and mounting. Microscopic images were acquired using a light microscope under identical lighting and exposure conditions. For semi-quantitative analysis, five randomly selected fields per section were captured at 400× magnification. The intensity of positive brown staining (DAB signal) was quantified using ImageJ software (version 1.54p, National Institutes of Health, USA). Briefly, the images were first converted to 8-bit grayscale, and a consistent threshold was applied to select the DAB-positive area across all images. The integrated density (product of area and mean gray value) was measured for each field. Background staining was subtracted by measuring the signal from a negative control region (secondary antibody only) on the same slide, and the net integrated density was used for statistical analysis. All quantifications were performed by an investigator blinded to the group allocation.

### 4.9. Bacterial DNA Extraction

Genomic DNA was extracted from fecal samples using a CTAB-based lysis method. The concentration and purity of the DNA were assessed spectrophotometrically by measuring the absorbance at 260 nm and calculating the 260/280 nm absorbance ratios, respectively. A total of 50 ng of high-quality genomic DNA was used as the template for PCR amplification. A total of 50 ng of high-quality genomic DNA served as the template for PCR amplification. Amplicons were purified with AMPure XP beads, quantified fluorometrically, and then used for Illumina-compatible library building.

### 4.10. 16S rRNA Sequencing and Data Processing

Library quality was assessed using an Agilent 2100 Bioanalyzer (Agilent Technologies, Santa Clara, CA, USA), and library quantification was performed using the KAPA Library Quantification Kit for Illumina platforms (KAPA Biosystems, Woburn, MA, USA). Qualified libraries underwent 2 × 250 bp paired-end sequencing on the NovaSeq 6000 platform. Following sequencing, samples were demultiplexed based on barcode information, and adapter and barcode sequences were removed. Sequences underwent assembly and quality filtering. Length trimming and denoising were performed using the DADA2 module within QIIME2 (dada2 denoise-paired), yielding ASV (feature) representative sequences and corresponding abundance matrices.

### 4.11. Bioinformatics Analysis

α-diversity was assessed based on the acquired ASV (feature) sequences and abundance tables. Taxonomic annotation was conducted utilizing the SILVA database with a confidence threshold of 0.7. Fisher’s exact test was employed for comparisons lacking biological duplicates; the Mann–Whitney U test was utilized for comparisons between two groups with biological replicates; and the Kruskal–Wallis test was applied for comparisons among multiple groups with biological replicates. Microbial community composition and correlation analyses were performed using the Microbiome Alliance platform [54], and heatmaps were generated using the Microbiome Informatics platform [55].

### 4.12. Metabolomics Sample Preparation

Fecal samples were thawed at 4 °C, and 100 mg of each sample was transferred into a tube containing three steel beads. Subsequently, 1000 μL of pre-cooled methanol–water solution (4:1, *v*/*v*) was added. The samples were homogenized at −10 °C for 5 min and then maintained at −20 °C for 30 min. An appropriate volume of the resulting supernatant was collected for LC-MS/MS analysis. Quality control (QC) samples were prepared by pooling equal aliquots from all individual samples and were used to equilibrate the chromatographic-mass spectrometric system and evaluate system stability throughout the analytical sequence [56,57].

Chromatographic separation was performed using a Shimadzu Nexera X2 LC-30AD ultrahigh-performance liquid chromatography system (Shimadzu Corporation, Kyoto, Japan) equipped with an ACQUITY UPLC HSS T3 column (2.1 × 100 mm, 1.8 μm; Waters, Milford, MA, USA). Samples were maintained at 4 °C in the autosampler. The injection volume was 16 μL, the column temperature was 40 °C, and the flow rate was 0.3 mL/min. Mobile phase A consisted of water containing 0.1% formic acid, whereas mobile phase B consisted of acetonitrile containing 0.1% formic acid. The gradient program was as follows: 0–2 min, 0% B; 2–6 min, 0–48% B; 6–10 min, 48–100% B; 10–12 min, 100% B; 12–12.1 min, 100–0% B; and 12.1–15 min, 0% B.

Mass spectrometric analysis was performed using a Q Exactive Plus mass spectrometer (Thermo Scientific) with electrospray ionization in both positive and negative ion modes. The HESI source parameters were as follows: spray voltage, 3.8 kV in positive mode and 3.2 kV in negative mode; capillary temperature, 320 °C; sheath gas, 30; auxiliary gas, 5; probe heater temperature, 350 °C; and S-lens RF level, 50. The mass spectrometric acquisition time was 15 min. Full-scan spectra were acquired over an *m*/*z* range of 75–1050 at a resolution of 70,000 at *m*/*z* 200, with an AGC target of 3 × 10^6^ and a maximum injection time of 100 ms. Data-dependent MS/MS acquisition was performed by selecting the ten most intense precursor ions after each full scan. The MS/MS resolution was 17,500 at *m*/*z* 200, with an AGC target of 1 × 10^5^, a maximum injection time of 50 ms, HCD activation, an isolation window of 2 *m*/*z*, and stepped normalized collision energies of 20, 30, and 40.

### 4.13. Metabolomics Data Processing and Bioinformatics Analysis

Raw LC-MS/MS data were processed using MS-DIAL version 4.9 for ion-peak extraction, peak alignment, retention-time correction, and peak-area extraction. Metabolite annotation was performed by matching accurate precursor masses with a mass tolerance of <10 ppm and MS/MS fragment ions with a mass tolerance of <0.01 Da against public databases, including the Human Metabolome Database (HMDB), MassBank, and Global Natural Products Social Molecular Networking (GNPS), as well as the in-house Bioprofile metabolite database (BP-DB). Ion peaks with more than 50% missing values within a group were excluded from subsequent statistical analysis. The positive- and negative-ion datasets were separately normalized to the total peak area and then integrated. The combined dataset was preprocessed using unit-variance scaling before subsequent analysis. Pattern recognition and multidimensional statistical analyses were performed using Python version 4.90. Quality control was evaluated using QC samples. After ion-peak extraction, a total of 69,766 ion peaks were obtained in positive- and negative-ion modes. Peaks extracted from all experimental and QC samples were subjected to PCA analysis after unit-variance scaling, and the PCA model was generated using sevenfold cross-validation.

### 4.14. Statistical Analysis

Statistical analyses were performed using GraphPad Prism 9. Continuous data are presented as the mean ± standard error of the mean (SEM), whereas categorical data are presented as percentages. Comparisons between two groups were performed using an unpaired *t*-test. Multiple-group comparisons were conducted using one-way ANOVA. Two-way ANOVA was used when two independent variables were included. For non-normally distributed data, the Kruskal–Wallis test was used for between-group comparisons. A *p* value < 0.05 was considered statistically significant.

## 5. Conclusions

This work illustrates that AR produces therapeutic benefits via complex interactions within the microbiota—metabolism—barrier axis. AR suppresses the proliferation of taxa such as *Ligilactobacillus*, *HT002*, and *Erysipelatoclostridium*, while facilitating the resurgence of taxa including *Bacteroides*, *Alloprevotella*, and *Lachnospiraceae_NK4A136_group*. Concurrently, AR regulates essential metabolites by downregulating 5OH-HIP while elevating 4-(2-Amino-3-hydroxyphenyl)-2,4-dioxobutanoate, vanillin, Isalexin, CHEBI:79180, and 3α-Hydroxy-3,5-dihydromonacolin L, with modifications in associated metabolic pathways. These modifications may diminish inflammatory triggers linked to dysbiosis, therefore enhancing intestinal homeostasis. Simultaneously, AR suppresses TLR4/TLR9-mediated inflammatory signaling, diminishes proinflammatory responses, and enhances the expression of the tight junction proteins OCC and ZO-1. These effects may improve intestinal barrier function, therefore breaking the detrimental loop of barrier disruption and heightened inflammation. These data collectively offer experimental evidence for AR as a viable remedy for IBD and indicate a potentially therapeutic benefit in illnesses related to gut inflammation.

## Figures and Tables

**Table 1:** Differentially expressed metabolites in the CON and DSS and AR-H group.

Metabolite Name	*m*/*z*	VIP	*p*	CON vs. DSS	DSS vs. AR-H
4-(2-Amino-3-hydroxyphenyl)-2,4-dioxobutanoate	222.0403	1.867036281	0.0000284	**↓**	**↑**
Vanillin	153.0546	1.830561054	0.0000194	**↓**	**↑**
Isalexin	313.2736	1.821792268	0.0000025	**↓**	**↑**
3alpha-Hydroxy-3,5-dihydromonacolin L	241.1434	1.64028096	0.0000017	**↑**	**↓**
CHEBI:79180	176.0329	1.394379421	0.0000044	**↓**	**↑**
5OH-HIP	339.2178	1.264666865	0.0000030	**↓**	**↑**

Note: ↑ indicates up-regulation and ↓ indicates down-regulation in the corresponding comparison groups.

**Table 2:** Correlations between gut microbiota and serum metabolites.

Genus	Metabolites	R	*p*
*Bacteroides*	4-(2-Amino-3-hydroxyphenyl)-2,4-dioxobutanoate	0.2244	0.3707
	Vanillin	0.4382	0.0689
	Isalexin	0.0069	0.9780
	3alpha-Hydroxy-3,5-dihydromonacolin L	0.7564	0.0003
	CHEBI:79180	0.6857	0.0017
	5OH-HIP	−0.5191	0.0273
*Alloprevotella*	4-(2-Amino-3-hydroxyphenyl)-2,4-dioxobutanoate	0.2241	0.3714
	Vanillin	0.4401	0.0676
	Isalexin	0.01029	0.9677
	3alpha-Hydroxy-3,5-dihydromonacolin L	0.7514	0.0003
	CHEBI:79180	0.6748	0.0021
	5OH-HIP	−0.4436	0.0652
*Ligilactobacillus*	4-(2-Amino-3-hydroxyphenyl)-2,4-dioxobutanoate	−0.2760	0.2676
	Vanillin	−0.3985	0.1015
	Isalexin	−0.1237	0.6250
	3alpha-Hydroxy-3,5-dihydromonacolin L	−0.2755	0.2685
	CHEBI:79180	−0.4433	0.0654
	5OH-HIP	0.2506	0.3158
*HT002*	4-(2-Amino-3-hydroxyphenyl)-2,4-dioxobutanoate	−0.5608	0.0155
	Vanillin	−0.5887	0.0102
	Isalexin	-0.5266	0.0248
	3alpha-Hydroxy-3,5-dihydromonacolin L	−0.4532	0.0589
	CHEBI:79180	−0.4230	0.0803
	5OH-HIP	0.6456	0.0038
*Erysipelatoclostridium*	4-(2-Amino-3-hydroxyphenyl)-2,4-dioxobutanoate	−0.4631	0.0529
	Vanillin	−0.5002	0.0345
	Isalexin	−0.5061	0.0321
	3alpha-Hydroxy-3,5-dihydromonacolin L	−0.3180	0.1984
	CHEBI:79180	−0.2265	0.3661
	5OH-HIP	0.1222	0.6292

## Data Availability

The research data can be found here: PRJNA1459665.

## Abbreviations

The following abbreviations are used in this manuscript:

AR	*Artemisia rupestris* water extract
IBD	Inflammatory bowel disease
DSS	Dextran sodium sulfate
DEX	Dexamethasone
IL-6	Interleukin-6
CXCL-1	C-X-C motif chemokine ligand 1
TNF-α	Tumor necrosis factor-α
MPO	Myeloperoxidase
TLR4	Toll-like receptor 4
TLR9	Toll-like receptor 9
OCC	Occludin
ZO-1	Zonula occludens-1
TJ	Tight junction
FITC	Fluorescein isothiocyanate
ELISA	Enzyme-linked immunosorbent assay
DAB	Diaminobenzidine
CTAB	Cetyltrimethylammonium bromide
PCR	Polymerase chain reaction
ASV	Amplicon sequence variant
QIIME2	Quantitative Insights Into Microbial Ecology 2
RDA	Redundancy analysis
LEfSe	Linear discriminant analysis Effect Size
LDA	Linear discriminant analysis
PLS-DA	Partial least squares discriminant analysis
UPLC-ESI-MS/MS	Ultra-performance liquid chromatography–electrospray ionization–tandem mass spectrometry
QC	Quality control
SCFAs	Short-chain fatty acids
GPR43	G protein-coupled receptor 43
AhR	Aryl hydrocarbon receptor
NF-κB	Nuclear factor kappa-B
ERK	Extracellular signal-regulated kinase
MyD88	Myeloid differentiation primary response 88
MAPK	Mitogen-activated protein kinase
MLCK	Myosin light-chain kinase
ROS	Reactive oxygen species
HOCl	Hypochlorous acid
PPARγ	Peroxisome proliferator-activated receptor gamma
FMT	Fecal microbiota transplantation
CON	Control
AR-L	Low-dose AR
AR-M	Medium-dose AR
AR-H	High-dose AR
DAI	Disease activity index
SEM	Standard error of the mean
